# Isolation, Purification, and Characterization of Homogenous Novel Bioactive Protein from *Datura stramonium* Stem Exhibited Larvicidal Activity against *Anopheles stephensi*

**DOI:** 10.1155/2022/1637896

**Published:** 2022-12-05

**Authors:** Manisha Kirar, S. P. Singh, Neelam Sehrawat

**Affiliations:** ^1^Department of Genetics, Maharshi Dayanand University, Rohtak 124001, Haryana, India; ^2^National Institute of Malarial Research, New Delhi, India

## Abstract

The insecticidal resistance of mosquitoes necessitates the development of a natural, safe, and plant-based method for vector control. Unfortunately, there are no effective vaccines or particular medications available to combat malaria; therefore, mosquitoes must be targeted directly. Previous studies have shown the health benefits of *Datura stramonium*, but its bioactive peptides or proteins are less explored. This is the first study on *D. stramonium* stem protein used for mosquito larval protein. The present study aimed to identify the purified mosquito larval protein from the crude extract of *D. stramonium* stem. Crude protein was isolated, precipitated, dialyzed, and purified by using ion-exchange chromatography, native PAGE, and HPLC. The highest larval mortality was observed at 5.5 mg/ml of crude protein concentration. Native PAGE was used for the analysis and purification of active proteins. The single homogeneous purified larvicidal protein appeared as a single band of 30 kDa by SDS-PAGE. The novel bioactive peptide was characterized by LC-MS/ESI-MS. The homology of the peptide was searched by the Mascot search engine. The database search revealed has not shown peptide similarity with *D. stramonium* protein, but homology with another plant Arabidopsis thaliana protein is probable for protein phosphatases. The lethal concentration of purified protein against 3^rd^ instar larvae of *Anopheles stephensi* had LC_50_ and LC_90_ values of 25 *μ*g/ml and 40 *μ*g/ml. It has shown new insight into larvicidal activity and can be used as a new drug against malaria and other mosquito-borne diseases.

## 1. Introduction

Spiders, mites, crabs, centipedes, millipedes, lobsters, and insects are all members of the Arthropod phylum, which is the largest in the animal kingdom. Mosquitoes belong to the insect family, and they are a well-known vector for a variety of disease-causing infections [1]. WHO has classified mosquitoes to be the number one public enemy [2]. Chikungunya, dengue fever, malaria, and filariasis are still major health problems in many countries. Mosquito-borne diseases are becoming epidemic diseases due to changing lifestyles and urbanization, resulting in the proliferation of larval habitats [3]. Humans suffer greatly as a result of these diseases. Malaria, leishmaniasis, yellow fever, Chagas disease, Japanese encephalitis, and trypanosomiasis are all vector-borne diseases that kill over 70,000 people each year [4]. Despite significant advances in the fight against malaria, an estimated 3.2 billion people, nearly half of the world's population, spread across 91 nations and territories remain at danger. Malaria claimed the lives of 409,000 people and sickened 229 million people in 2019 [5].

*D. stramonium* is a blooming plant that is tall, annual, and branched and belongs to the Solanaceae family. *Datura* is found in ten species, however, only two of them, *Datura innoxia* and *D. stramonium*, have known drug-like effects [6]. The plant has a role as insect repellent, antioxidant, antimicrobial, anticancer, and anti-inflammatory agents, and larvicidal and mosquito repellent and has anticholinergic activity [7]. The plant contains alkaloids, steroids, glycosides, tannin, flavonoids, saponin, atropine, phenol, protein, carbohydrates, and fat, according to the phytochemical analysis [8]. *Datura stramonium* ethanolic leaf extract possesses larvicidal and repellent actions against *Anopheles stephensi* and *Culex quinquefasciatus* [9]. Plants have been employed for the treatment of human diseases throughout the world since ancient times. Synthetic pesticides have the potential to affect water, soil, and the environment. Botanical pesticides are safe, less toxic, effective, affordable, and environmentally friendly [10]. Mosquitoes are effectively controlled by proteins derived from several plants [11–13]. The various poisonous proteins (lectin, ricin, RIPs, alpha amylase inhibitors, PIs) are found in plants and have insecticidal activities against various insects [14]. A brief literature study indicated that there are not many studies regarding the isolated, characterization and purification of antilarvae proteins from plants so that the *D. stramonium* stem protein has to be characterized by its specific larvicidal activity.

The present study has been carried out to analyse the larvicidal potential of the stem of *D. stramonium* protein. The crude protein was isolated from the stem of D. stramonium by the TBS buffer and ammonium sulphate precipitation. The protein extract was subjected to a mosquito larvicidal bioassay according to WHO guidelines. The crude protein was subjected to DEAE-cellulose column chromatography and native PAGE for purification. HPLC and SDS-PAGE were also performed to check the purity of the extract. The native PAGE purified protein was trypsin digested and identified by the LC/MS. The peptides obtained through the LC/MS were searched and identified with the help of the Mascot search engine.

## 2. Materials and Methods

### 2.1. Collection and Identification of Plant Sample

The plant part has been collected from the herbal garden of M. D. U., Rohtak, Haryana. The identification of plant material has been done by plant taxonomist Dr. Surender Yadav, Assistant Professor, Department of Botany, M. D. U., Rohtak, Haryana.

### 2.2. Mosquito Rearing

Larvae of *An. stephensi* were procured from NIMR, New Delhi. The culture of the mosquito was maintained at (26 ± 2)°C with a photoperiod of 12 : 12 h (light:dark) in the insectory of the Center for Biotechnology, M.D.U., Rohtak, and the larvae were fed with dog food. The pupae were transferred in a small plastic bowl and kept in a mosquito cage for adult emergence. The cotton pads were soaked in 10% aqueous glucose solution and kept in cages for mosquito feeding. The rabbit was put in the cage for mosquito blood-feeding. For mosquito egg laying, a plastic bowel containing filter paper on the boundaries immersed with water was kept in the cage.

### 2.3. Optimization of Different Buffers to Obtain Higher Yield of Protein

Total protein was extracted by the use of different buffers, (a) phosphate buffer (50 mM, pH 7.8); (b) sodium acetate buffer (50 mM, pH 5.5); (c) Tris-buffer (0.1 M, pH 7.5); (d) Tris-buffer saline (50 mM, pH 7.5), to obtain higher yield of protein.

### 2.4. Protein Extraction from Stem

The collected plant part was first washed with tap water and then with distilled water and kept for shaded dry at room temperature. The sample was powdered with the help of an electric grinder and liquid nitrogen. The total protein was extracted by the use of extraction buffer Tris-buffer saline (50 mM Tris-HCl (pH, 7.5), 150 mM NaCl, and polyvinyl pyrrolidine) with a slight modification in the ratio of the extraction buffer. Total protein was isolated in an extraction buffer in the ratio of 1 : 7 (w/v). The sample was filtered with 2 –3 layers of muslin cloth and the filtrate sample was kept on a magnetic stirer at 4° C overnight. The sample was centrifuged at 13,000 rpm for 30 minutes at 4°C. The supernatant was collected and the pellet was discarded.

### 2.5. Protein Precipitation

The crude protein extract was obtained through the Tris-buffer saline and precipitated by different saturation percentages initially 20%, 30–40%, 50– 60% ,70–80%, and 90–100% of ammonium sulphate. The 80% saturated ammonium sulphate solution has given a good quality pellet after centrifugation at 13,000 for 30 min at 4°C. The supernatant was precipitated with ammonium sulphate by 80% saturation, and the sample was kept at −20°C overnight to completely precipitate the protein. The next morning sample was centrifuged at 14,000 rpm for 30 minutes at 4°C, the supernatant was discarded, and the pellet was washed 5 –6 times with acetone and the sample was dried. The protein sample was dialyzed against distilled water for 24 h at 4°C. The protein sample was kept at −20°C for further bioassay and purification.

### 2.6. Protein Quantification

The total protein concentration of the isolated sample was calculated by the standard Bradford method by UV spectrophotometer at 595 wavelength [15]. BSA was taken as a standard stock solution of 1 mg/ml.

### 2.7. Larvicidal Bioassay

Larvicidal bioassay was conducted using the third instar larvae of *An. stephensi* according to the guidelines of the WHO [16]. Ten larvae of the 3^rd^ instar stage were placed in a plastic bowl containing water (99 ml) and test solution (1 ml), with a final volume of 100 ml. Six different concentrations of crude protein extract (0.172, 0.34, 0.68, 1.37, 2.75, and 5.5 mg/ml) and purified protein (10, 20, 30, 40, 50, and 60 *μ*g/ml) were prepared from the stock solution of protein extract with distilled water and used for the bioassay. Larvae mortality was monitored after 24, 48, and 72 hours. A control was set up with Tris-buffer saline. The experiment was conducted in triplicate.

### 2.8. DEAE-Cellulose Column Chromatographic Separation

The protein was purified by IEC (ion exchange chromatography). The dialyzed protein sample showing relatively higher larvicidal activity was subjected to purification using ion-exchange chromatography by passing the protein through the DEAE-cellulose column. Lyophilized protein samples were dissolved in Tris-HCl (50 mM; pH 7.5) and loaded onto a DEAE-cellulose column at the flow rate of 0.5 ml/min. The unbound proteins were eluted with Tris-HCl (50 mM; pH 7.5), and the bound proteins were desorbed with the same buffer with a gradient of NaCl (0.0–0.5 M). The unbound and bound proteins were collected in sterile tubes. A different number of fractions were collected from all samples, and they were tested for larvicidal activity using the WHO protocol bioassay. Those fractions that showed larvicidal activity were pooled together and dialyzed against a Tris-HCl buffer. The dialyzed samples were lyophilized and stored at −20°C and further analyzed for the larvicidal test.

### 2.9. Preparative Native PAGE

The protein fractions obtained from DEAE-cellulose column chromatography having the highest larvicidal activity were analyzed by preparative native PAGE [17]. Electrophoresis was carried out on Bio-Rad gel plates by using 5% stacking gel and 12% resolving gel. The test samples were solubilized in sample buffer and loaded to the well of gel, and electric current of 50 V was firstly applied and then a supply of 150 V. When the dye reached the bottom, power supply was switched off, and the gel was put in Coomassie Brilliant Blue G-250 staining solution at 4°C overnight with shaking. In the morning, the gel was destained and we visualized the bands. The standard protein marker was used to determine the molecular weight of the test samples. The protein band of a small portion was cut out from the gel with the help of a sterile blade and kept in a destaining solution for complete removal of the dye. The protein was eluted from the gel by grinding in a chilled mortar and pestle, and the gel slurry was tied to a dialysis membrane. The dialysis membrane was immersed in a native PAGE buffer and run for 1 h. The sample was collected from the membrane and centrifuged at 12,500 rpm for 30 min at 4°C. The protein was present in the supernatant and used for purity and larvicidal bioassay.

### 2.10. HPLC Analysis

The homogeneity of the purified protein was analyzed by using Agilent 1100 High Performance Liquid Chromatography (HPLC) (Agilent Technologies, USA) on a C18 column (USCFX03064 EC-C18, 2.7 *μ*m, 3.0 × 100 mm, USA) as described by Schwarz [18]. The 60 *μ*g of the sample was prepared after mixing with its DDT, methanol, and MS grade water. The sample was incubated for 30 minutes in the dark and loaded onto the HPLC. The peak was observed at the retention time of min exactly coincided with that of the Tris-HCl buffer in which the protein was originally dissolved. The chromatogram of the sample along with the blank was used for analysis.

### 2.11. Molecular Mass Determination by SDS-PAGE

The purified protein was electrophoresed on SDS-PAGE gel along with a protein standard molecular weight marker (Bio-Rad, USA). The molecular weight of the purified protein was determined by staining with Coomassie Brilliant Blue and comparing the band along with a standard protein marker.

### 2.12. LC-MS/ESI-MS Analysis

The LC-MS/ESI-MS (ESI-QUAD-TOF) analysis was performed to confirm the purity of the protein. The purified protein band was cut out from the gel with the help of a sterile surgical blade and put into distaining solution and subjected to trypsin digestion. The trypsin digested peptides were identified by LC-MS/ESI-MS.

### 2.13. Protein Identification

By MASCOT search engine (Matrix Science), peptide match and identification of peptide were performed. MS and MS/MS data were submitted to the MASCOT search program (https://www.matrixscience.com/). The search was also performed using the Swiss-Prot and NCBI database, restricted to Viridiplantae (green plants). The search criteria were established considering carboxymethyl (C) modifications as fixed effects and the alteration of the oxidation of the methionines as a variable effect. In trypsin hydrolysis, the possible loss of a cleavage site was considered, and the tolerance of the peptide and fragment masses was ±0.3 Da.

### 2.14. Statistical Analysis

The data were subjected to probit analysis to calculate the LC_50_, LC_90_, 95% confidence limit, and *R*-square value. All the experiments were carried out in triplicate. Microsoft Excel version 2007 software was used for the statistical analysis. The mortality rate was corrected with the help of Abbott's correction formula [19].

## 3. Results

### 3.1. Optimization of Different Buffers to Higher Yield of Protein

Total protein was extracted by the use of different buffers: (a) phosphate buffer (50 mM, pH 7.8), (b) sodium acetate buffer (50 mM, pH 5.5), (c) Tris-buffer (0.1 M, pH 7.5), and (d) Tris-buffer saline (50 mM, pH 7.5), as shown in Table 1. But a higher percentage of yield was obtained in the extraction with Tris-buffer saline. Then, the protein was isolated with the use of Tris-buffer saline extraction buffer from the stem of *D. stramonium*.

### 3.2. Screening of Larvicidal Protein from the Extract of Plant Stem

**Figure 1:**
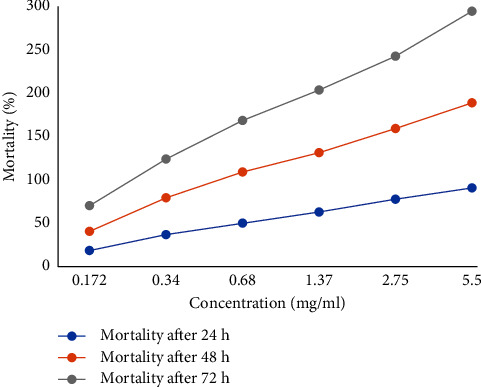
Mortality percentage in *D. stramonium* crude protein stem extract at different concentrations of protein solution.

**Figure 2:**
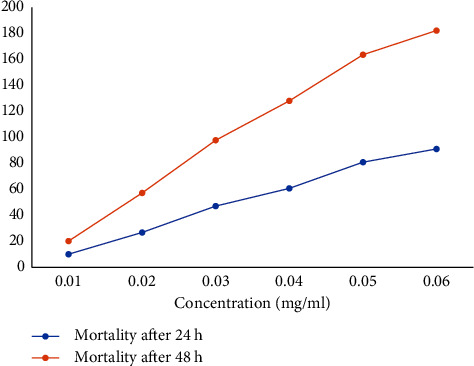
Mortality percentage in *D. stramonium* purified protein at different concentrations of protein solution.

Protein extracted from the stem of *D. stramonium* was screened for mosquito larvicidal activity using the WHO protocol against 3^rd^ instar larvae of *An. stephensi*. The first step of purification was done by precipitation with ammonium sulphate, and protein fractionated with 70–80% ammonium sulphate was taken for testing the larvicidal activity. The partially purified ammonium sulphate extract and dialyzed protein showed 80–90% mortality at the concentration of 5.5 mg/ml after 48 h.  Table 2 shows mortality percentage at different concentrations after serial dilution from 0.172 to 5.5 mg/ml of crude protein extract and purified protein at different concentrations (10 to 60) *μ*g/ml. Larvae mortality at different concentrations of protein are shown graphically in Figures 1 and 2. The LC_50_ and LC_90_ values of plant proteins for the third instar larvae of *An. stephensi* after 48 h of exposure are shown in Table 3. The rate of mortality is directly proportional to increased concentration to dose. A correlation exists between the saturation of precipitation and the molecular mass of the proteins. Lesser saturation precipitates a higher molecular mass of protein and higher saturation precipitates the low molecular mass proteins. In the present study, higher larvicidal activity was observed at a crude protein extract concentration of 5.5 mg/ml. The mortality rate was corrected with the help of Abbott's correction formula. The LC_50_ and LC_90_ values of plant proteins for the third instar larvae of *An. stephensi* after 72 h of exposure are shown in Table 3.

### 3.3. Purification of Protein by DEAE-Cellulose Column Chromatography

**Figure 3:**
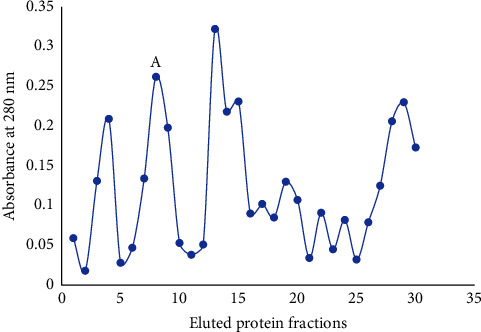
Elution profile of *D. stramonium* stem protein obtained from DEAE-cellulose column.

The crude protein extract showing the highest mortality was used for further purification on DEAE-cellulose ion (anion) exchanger chromatography. A total of 35 fractions were collected of 1 ml sample each as shown in Figure 3, and each fraction was tested for larvicidal bioassay using the WHO protocol. The percentage larval mortality of 20%, 10%, 25%, 10%, 30%, and 25% was observed in different eluted fraction numbers 6–11. The active fractions 6–11 were pooled together and dialyzed against TBS (pH 7.5) and lyophilized. The percentage of protein yield of chromatography samples was obtained at 4.2% as shown in Table 4. The pooled active protein fraction A was showing 90% larvicidal potential at a concentration of 60 *μ*g/ml as shown in Figure 2. The protein fraction A has a single peak as shown in Figure 3, which clearly reveals that the stem of *D. stramonium* has only one larvicidal protein. Further purification was performed to confirm the presence of homogenous protein from eluted fractions.

### 3.4. Purification of Larvicidal Protein by Preparative Native Page

**Figure 4:**
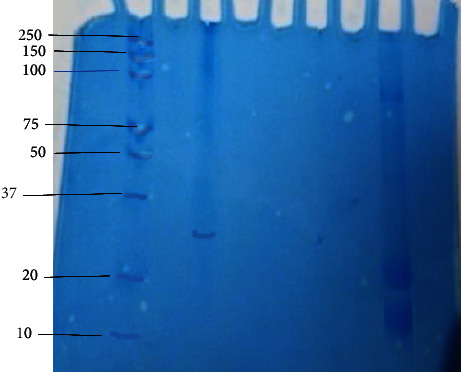
Native PAGE analysis of antilarval protein from the stem of *D. stramonium*. Lane 1, protein standard marker (10–250 kDa). Lane 2, purified protein from DEAE-cellulose chromatography and native PAGE. Lane 3, crude protein extract.

The native page was done to analyse the presence of crude protein and purified protein present in the stem of *D. stramonium*. The single protein band was observed from the native page gel, as shown in Figure 4. This purified single protein has larvicidal activity, and this band was eluted from the gel by the electrodialysis method. The total percentage of yield is 2.2% obtained by preparative native PAGE, as shown in Table 4. The purified protein was used for the larvicidal bioassay against the larvae *An. stephensi* and further for molecular characterization. The molecular mass of the purified protein was calculated by extrapolating the mobility value (*Rf* value) of the purified protein with the relative mobility values of the standard molecular mass protein (Figure 4).

### 3.5. HPLC Analysis

**Figure 5:**
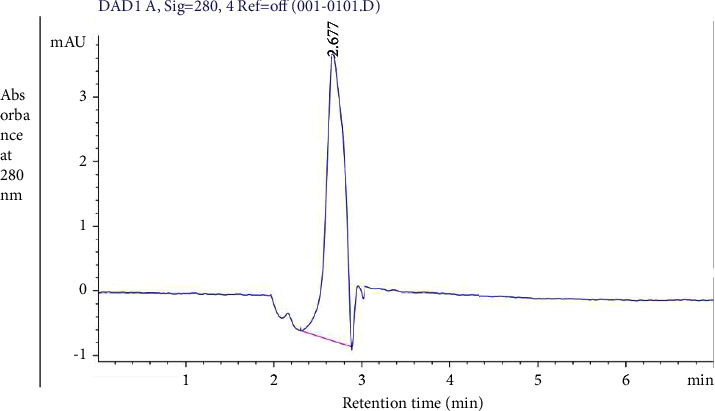
HPLC analysis of *Datura stramonium* purified stem protein shows that one peak of retention time is 2.677 at 280 nm.

HPLC is a technique used to separate a single compound from a mixture of components based on peak analysis. The homogeneity of the purified protein was analyzed using HPLC and peak purity analysis, whereas the single peak observed belonged to the purified protein with a high purity index. It showed only a single peak, confirming that the protein is 100% pure and without any impurity. The chromatogram showed two peaks with one peak at a retention time of min representing the presence of purified protein and another peak at a retention time of min representing the Tris-buffer saline in which the protein was dissolved. The chromatogram clearly indicates the presence of a single peak that confirmed the protein is 100% pure (Figure 5).

### 3.6. Mass Determination by SDS-PAGE

**Figure 6:**
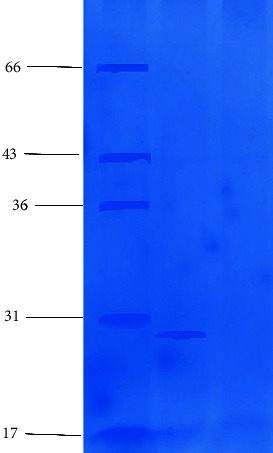
SDS-PAGE of purified protein (17–66 kDa). Lane1, protein standard marker. Lane 2, purified antilarval protein.

SDS-PAGE reveals that a single homogenous band was appeared on the gel. The molecular mass of the purified protein was determined as 30 kDa (Figure 6).

### 3.7. LC-MS/MS Analysis

**Figure 7:**
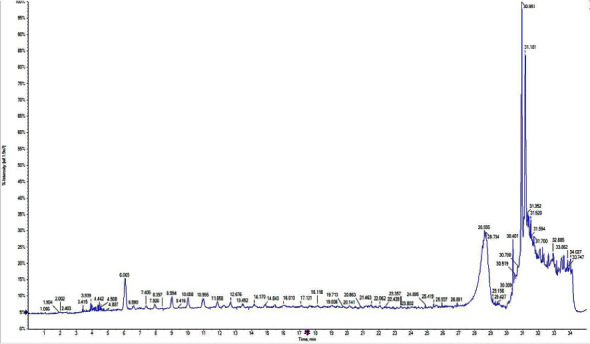
LC-MS/ESI-MS spectra of purified protein of *D. stramonium*.

The purified protein isolated from D*. stramonium* was digested with trypsin, and the peptides obtained were subjected to LC-MS/ESI-MS analysis. The mass spectra (Figure 7) was searched by matching with the MASCOT search engine, and the peptide was identified as shown in the Table 5. The purified peptide does not show similarity with the *D. stramonium* protein. The purified peptide (R.LVAKAAAR.A) showed similarity with a phosphatase protein of *Arabidopsis thaliana* as shown in Table 5. The protein NCBI blast also showed the maximum similarity with the phosphatase protein of *Arabidopsis thaliana*. The complete genome sequence of *D. stramonium* with annotated information is not available till now. This could be as certain as the reason for their lack of similarity.

## 4. Discussion

The search for larvae protein from botanical sources is a new approach to combat the vector control and the formulation of protein-based drugs. Plant proteins have been reported in many biological activities, including antibacterial, antilarvicidal, and antiviral properties [20–22]. The mull protein was purified from *Myracrodruon urundeuva* leaf, and it exhibited LC_50_ value of 0.202 mg/ml against *Aedes aegypti* larvae [23]. The type IInd RIP from the camphor seed (*Cinnamomum camphora*) has LC_50_ value 168 ppm against the larvae of *Culex pipines pallens* [24]. The biological effect of ApTI protein on *Aedes aegypti* larvae was decrease in survival rate after 96 h of treatment, from 93.08 ± 5.01% to 69.22 ± 10.88%, respectively. For 1 mg/ml ApTI, 100% mortality was observed, while the mortality of the control group reached a maximum of 10% [25]. One approach to reduce the mosquito population involves interrupting the mosquito's life cycle at the larval stage [26]. Synthetic larvicides in comparison to the natural larvicides are harmful to aquatic organisms and the environment due to their hydrophobic nature. *Anopheles stephensi* has been reported susceptible to temephos in India with LC_50_ range of 0.008–0.015 ppm [27–29]. During the last three decades, temephos, an organophosphate compound, has been considered as a safe larvicide (LC_50_ = 8600 mg/l) in vector control programs [30]. There are reports of resistance to some insecticides such as DDT, diledrin, and malathion in *An. stephensi*, as well as some indications of resistance to pyrethroids in the current years [31, 32]. Another study on temephos reveals the LC_50_ value of 0.0523 ppm and LC_90_ value of 0.3822 ppm for the *An. stephensi* [33]. WHO suggested a temephos dose of 1 ppm for larviciding in water bodies. Our results have LC_50_ and LC_90_ values of 20 *μ*g/ml and 40 *μ*g/ml of purified protein against *An. stephensi* larvae, which is an acceptable amount of dose according to other larvicide compounds. Swathi et al., in 2012, reported that the ethanolic extract of *Datura stramonium* leaves showed LD_50_ (86.2518 ppm) and LD_90_ (196.389 ppm) against *Aedes aegypti*, LD_50_ (16.0783 ppm) and LD_90_ (41.9599 ppm) against *Anopheles stephensi,,* and LD_50_ (6.25 ppm) and LD_90_ (11.25 ppm) against *Culex quinquefasciatus*. The root extract of *D. stramonium* has 100% mortality after 24 h of treatment at 100% concentration [34]. *Datura stramonium* and *Nicotiana tabaccum* have high larvicidal properties and can be used as environmentally friendly and sustainable insecticides to control mosquitos [35]. The ethanolic leaf extract of *D. stramonium* has LC_50_ 3.29 ppm and LC_90_ 55.25 and n-hexane had lower LC_50_ 2.52 ppm and LC_90_ value was 30.51 ppm against *C. quenquifaciatus* larvae. The toxicity of fraction DSEE-F1 of the leaf extract of *D. stramonium* showed a maximum activity of 99% at 100 ppm after 24 hr against *C. quinquefasciatus*, the third and fourth instar larvae [36]. Effective mortality was found at 93% at 25 ppm after 24 h of treatment with the lowest LC_50_ and LC_90_ values of 4.390 ppm and 6.957 ppm, respectively. A novel 20.9 kDa antimicrobial protein was purified from the seedling of *Bauhinia purpurea* and exhibited a potent antimicrobial activity against *Bacillus cereus* and *Escherichia coli*. The MIC (minimum inhibitory concentration) of this purified protein was 13 *μ*g/ml and 15 *μ*g/ml against both Gram-positive and Gram-negative bacteria [37]. The protein purified and identified from the native Amazonian species exhibited antifungal activity [38]. In the present study, proteins have been isolated, purified, and characterized from the stem of *D. stramonium* that exhibited larvicidal activity against *An. stephensi*. The purified protein showed 90% mortality at 40 *μ*g/ml. A single band of 30 kDa was shown by SDS-PAGE gel. HPLC single peak result revealed that the protein was in a homogenous stage. The purified protein was subjected for trypsin digestion and identified by LC-MS/MS. The protein identity and homology were checked by the Mascot search engine. The purified protein does not show homology with the *D. stramonium* protein. The homology of protein was matched with the protein phosphatase of plant *Arabidopsis thaliana*. Hence, the novel peptide was identified in the stem of *D. stramonium*. The LC_50_ and LC_90_ values of the purified protein were 0.025 and 0.04 mg/ml. Plant protein phosphatases have catalytic activity by dephosphorylation of the phosphoprotein [39]. Phosphate groups are important in activating proteins so that the proteins can perform particular functions in cells. Plant protein phosphatases have a role in stress signalling and defence mechanism against pathogens, but the complete study of their mechanism is still unknown [40]. Larvicidal activity of isolated proteins could be because of dephosphorylation of mosquito enzymes responsible for metabolic activities, immunity, survival, etc.

## 5. Conclusion

In the previous study, *D. stramonium* plant extract had mosquito, larvicidal, and repellence activity against *An. stephensi*, *Aedes aegypti,* and *Culex quinquefasciatus* at low concentration of lethal dose. This is the first report on the study of *D. stramonium* protein used against larvae of *An. stephensi*. The present study showed that the purified protein phosphatase of *D. stramonium* is able to kill the mosquito larvae of *An. stephensi*. The mechanism of activity of the phosphatase protein may involve the dephosphorylation of mosquito enzymes that kill the larvae. The purified protein has a 90% mortality at a low dose of LC_50_ 25 *μ*g/ml and LC_90_ 40 *μ*g/ml. The present study identified a novel larvicidal protein of 30 kDa from *D. stramonium* stem. This protein can be used in the formulation of safe, natural, plant-based mosquitocides for the control of mosquito-borne diseases without unbalancing the ecosystem. Further studies are also required to check the enzymatic activities of this isolated protein.

## Figures and Tables

**Table 1:** Percentage of protein yield obtained through different extraction buffer.

S. no.	Buffer used	Total amount (mg)	Yield % in crude sample
1	Phosphate buffer	4000	80
2	Sodium acetate	3250	65
3	Tris-HCl	4250	85
4	Tris-buffer saline	4800	96

**Table 2:** Percentage mortality of 3^rd^ instar larvae of *An. stephensi* at different concentrations of dialyzed crude and purified protein extract.

Protein extract	Concentration (mg/ml)	Period of exposure (hrs)		
		24 h ± S.D.	48 h ± S.D.	72 h ± S.D.
Crude protein	0.172	20 ± 5	20 ± 5	26.6 ± 5.8
	0.34	36.6 ± 4.0	38.3 ± 3.5	40 ± 0.0
	0.68	48.3 ± 2.9	53.3 ± 2.7	53.3 ± 2.8
	1.37	60 ± 0.0	61.6 ± 3.5	65 ± 0.0
	2.75	73.3 ± 2.9	73.3 ± 2.8	75 ± 0.0
	5.5	85 ± 3.5	88.3 ± 3.2	95 ± 0.0

Purified protein	0.01	10 ± 0.0	10 ± 0.0	
	0.02	26.6 ± 3.19	30 ± 0.0	
	0.03	46.6 ± 7.02	50 ± 0.0	
	0.04	60 ± 0.0	66.6 ± 8.16	
	0.05	80 ± 0.0	81.6 ± 4.08	
	0.06	90 ± 0.0	90 ± 0.0	

**Table 3:** The LC_50_ and LC_90_ values of crude and purified plant proteins against third instar larvae of *An. stephensi*.

Mosquito species	Protein extract	LC_50_ (mg/ml)	LC_90_ (mg/ml)	Regression equation	*R* ^2^	95% confidence intervals (LCL–UCL)
*An. stephens*i	Crude protein	0.489	5.43	*Y* = 0.0221*x* + 4.259	0.896	5.08–5.68
	Purified protein	0.025	0.04	*Y* = 0.0391*x* + 3.357	0.9534	1.73–6.28

*R* = coefficient of regression equations; LC_50_ = lethal concentration that kills 50% of larvae; LC_90_ = lethal concentration that kills 90% of larvae.

**Table 4:** Purification of mosquito larvicidal protein from the stem of *D. stramonium*.

S. no.	Name of sample	Total amount of protein (mg)	Yield (%)
1	Crude extract	650	100
2	Ammonium sulphate precipitation	150.5	40
3	DEAE-cellulose column	12	4.2
4	Preparative native PAGE	7.8	2.2

**Table 5:** Peptide mass fingerprinting subunit analysis of purified protein of *D. stramonium*.

P2C40ARATH mass: 54150 score: 40 expect: 0.023 matches: 1 Rec name: full = probable protein phosphatases 2C							
Observed	Mr (expt)	Mr (calc)	Delta	Miss	Rank	Unique	Peptide

400.2554	798.4963	798.5075	−0.0112	1	1	U	R. LVAKAAAR.A
PSD1B_ARATH mass: 108866 score: 30 expect: 0.024 matches: 1 26S proteasome non-ATPase regulatory subunit 1 homolog B							

Observed	Mr (expt)	Mr (calc)	Delta	Miss	Rank	Unique	Peptide
400.2554	798.4963	798.4963	0.0000	0	0.024	U	R. LVAIVER.M

## Data Availability

The data will be available on valid request to the corresponding author.
